# Progression of Bone and Joint Destruction During the Perinatal Period in Patients With Rheumatoid Arthritis and Juvenile Idiopathic Arthritis in the Last Decade

**DOI:** 10.7759/cureus.25396

**Published:** 2022-05-27

**Authors:** Takaaki Nagase, Yuya Takakubo, Yuki Yokoyama, Saeko Nagase, Suran Yang, Ryusuke Honma, Hiroharu Oki, Juji Ito, Akiko Sasaki, Michiaki Takagi

**Affiliations:** 1 Orthopaedic Surgery, Yamagata University Faculty of Medicine, Yamagata, JPN; 2 Orthopaedics and Rehabilitation, Yamagata University Faculty of Medicine, Yamagata, JPN; 3 Orthopaedic Surgery, Yamagata Saisei Hospital, Yamagata, JPN

**Keywords:** joint destruction, modified total sharp score, perinatal period, pregnancy, rheumatoid arthritis

## Abstract

Background: Rheumatoid arthritis (RA) is a disease that can cause joint destruction and multiple arthritis. We retrospectively investigated bone and joint destruction during the perinatal period in adult patients with RA and juvenile idiopathic arthritis (JIA) in our hospitals in the last decade.

Methods: The study included 15 women, with 20 pregnancies, 19 childbirths, and one fetal death recorded between 2009 and 2018. We analyzed patient characteristics, disease activity, the modified total Sharp score (mTSS), and ΔmTSS from prepregnancy to delivery and from delivery to one year after delivery in the biologics (BIO) group (biologics used before pregnancy) and non-BIO group (not using biologics).

Results: There were five preterm births and seven low-birth-weight infants. The Clinical Disease Activity Index (CDAI) before pregnancy and postdelivery worsened from 12±1.8 to 19.9±2.7 (*p*<0.05). The mTSS at prepregnancy and postdelivery was 47.7±12.2 and 57.3±11.1 in the BIO group, respectively, and 58.9±11.9 and 75.0±13.1 in the non-BIO group, respectively. In addition, the ΔmTSS value from prepregnancy to delivery and from delivery to one year after delivery was 14.5±4.8 and 9.2±1.7 in the BIO group, respectively (*p*<0.05), and 16.1±5.2 and 8.3±4.0 in the non-BIO group, respectively.

Conclusion: The disease activity worsened, and bone and joint destruction progressed in both the BIO and non-BIO groups during the perinatal period in adult patients with RA and JIA in the last decade.

## Introduction

Rheumatoid arthritis (RA) is a disease that can cause joint destruction and multiple arthritis. The prevalence is reported to be 0.6%-1.0% [1]. Synovitis occurs in the joint and gradually invades multiple joints throughout the body. Joint destruction, deformity and instability of the joint induce functional disorder of the joint. Rheumatoid arthritis often occurs in women aged 30-50, which is consistent with reproductive age [2-4]. The disease activity of RA affects pregnancy and childbirth. Although past reports indicate that RA symptoms of arthritis improve during pregnancy, that rate is only 16%-27% [5,6].

Several drugs are recommended to be withdrawn during the perinatal period of adult patients with RA and juvenile idiopathic arthritis (JIA). Of these drugs, methotrexate (MTX) is associated with increased miscarriage, fetal death and teratogenicity; nonsteroidal anti-inflammatory drugs (NSAIDs) have been reported to have a risk of pulmonary hypertension due to early arterial canal closure, and leflunomide and mizoribine have been reported to have a risk of teratogenicity [2,3,7-9]. However, withdrawal of antirheumatic drugs for patients with RA leads to worse disease activity [7,10]. When the disease activity of RA is active during pregnancy, it may adversely affect pregnancy complications and continuity of pregnancy and cause bone and joint destruction by severe arthritis [3,4,9].

The European Alliance of Associations for Rheumatology (EULAR) recommendation for the use of antirheumatic drugs in pregnancy published in 2016 indicates that hydroxychloroquine, salazosulfapyridine (SASP), azathioprine, cyclosporine, tacrolimus (TAC), and colchicine are safe for pregnant women with RA. It is recommended to continue these drugs during pregnancy to maintain remission [3,4,9,10].

In Japan, the expert committee of the Ministry of Health Labor and Welfare published guidelines in 2018, indicating that SASP, TAC, mercaptopurine, hydroxychloroquine, and antibody preparations are not teratogenic at present and can be administered to a pregnant woman with rheumatic disease [9]. In addition, reports of the safety and effectiveness of tumor necrosis factor (TNF) inhibitors during pregnancy have increased in the last decade [7,10,11].

Maintaining low RA disease activity and preventing bone and joint destruction progression using those drugs are important to maintain pregnancy. To the best of our knowledge, there are no reports estimating bone joint destruction in a pregnant woman with RA during the perinatal period. In this study, we retrospectively investigated bone and joint destruction in perinatal adult patients with RA and JIA over the past 10 years.

## Materials and methods

We had 34 pregnancies in 18 women, out of whom 13 with RA and 5 with JIA were pregnant and gave birth in our three hospitals from 2009 to 2018. Of 34 pregnancies in 16 women, this study retrospectively analyzed 20 pregnancies in 15 women as their X-ray images with their prepregnancy medical records and their postdelivery records were available to estimate their modified total Sharp score (mTSS) and clinical features. Patients with RA and JIA had to fulfill the revised 1987 American College of Rheumatology (ACR) classification or the 2010 ACR/EULAR classification criteria for RA, and the 2004 International League of Associations for Rheumatology classification of JIA [12-14]. Of 20 pregnancies, 13 pregnancies in 9 women were included in the biologics (BIO) group (biologics used before pregnancy), and 7 pregnancies in 6 women were included in the non-BIO group (not using biologics). All patients were treated by rheumatologists in our departments. Survey items included age at pregnancy, disease duration, C-reactive protein (CRP) level, Clinical Disease Activity Index (CDAI), medication for RA, adverse events during the perinatal period and ΔmTSS from prepregnancy to delivery and from delivery to one year after, which was evaluated by two independent orthopaedic surgeons (TN and YT). The interobserver variability for ΔmTSS was 90% (κ=0.43), and the intraobserver variability for evaluation was 94% (κ=0.48). The treatment strategy of our departments in 2009 allowed pregnancy following two “menstrual periods” after withdrawing MTX, in case of using MTX. Although prednisolone (PSL) only was allowed to continue, BIO and all conventional synthetic disease-modifying antirheumatic drugs (csDMARDs) were discontinued. After 2016, we changed our strategy regarding the use of BIO and csDMARDs with the pregnant patient’s consent when there were more benefits for the mother and fetus due to maintaining remission or low disease activity of RA compared to the withdrawal of the drugs.

Mann-Whitney U test analysis was performed using IBM® SPSS® Statistics, version 25 (IBM Corp., Armonk, NY). The study protocol adhered to the principles of the Declaration of Helsinki 2013 and was approved by the Ethics Committee of Yamagata University (2019-20).

## Results

Our study included 15 pregnant women with 20 pregnancies, 19 childbirths and 1 fetal death, consisting of 11 RA and 4 JIA patients. The mean age at pregnancy was 31.7 years (range 25-39), and the mean disease duration of RA and JIA was 7.2 years (range 0.6-20; Table 1). There were five preterm births (average 35.4 weeks, range 33 weeks 6 days to 36 weeks) and seven low-birth-weight infants (average 2252 g, range 1990-2406 g; Table 2). The CDAI before pregnancy and before delivery worsened from 9.3±1.8 to 19.9±2.8 in the BIO group (*p*<0.05), 16.0±3.8 to 17.9±2.4 in the non-BIO group, and from 12±1.8 to 19.2±2.7, in total (*p*<0.05; Table 3). There were 13 pregnancies in the BIO group and 7 pregnancies in the non-BIO group. After confirming pregnancy, biologics were stopped in all cases, and PSL was administered or maintained in nine of the patients (mean dosage, 8.8 mg/day, range 5-18) in the BIO group (administered in eight patients before pregnancy and one patient after pregnancy). csDMARDs were stopped in all cases, and PSL was administered for three of the patients (mean dosage, 7.0 mg/day, range 5-10) in the non-BIO group (all patients administered before pregnancy). In this study, adalimumab (ADA), etanercept (ETN) and tocilizumab (TCZ) were used as biologics (ADA in 1 patient, ETN in 10 patients, and TCZ in 2). Bucillamine (BUC) and SASP were used as csDMARDs (BUC in 2 and SASP in 5).

**Table 1:** Characteristics of the pregnant patients in BIO and non-BIO groups with RA and medication in the perinatal period RA: rheumatoid arthritis, JIA: juvenile idiopathic arthritis, BIO: biologics, PSL: prednisolone, NSAID: non-steroidal anti-inflammatory drug, ADA: adalimumab, ETN: etanercept, TCZ: tocilizumab, SASP: salazosulfapyridine, BUC: bucillamine, MTX: methotrexate, csDMARD: conventional synthetic disease-modifying antirheumatic drug, CZP: certolizumab pegol In the BIO group, biologics and/or csDMRADs were used before pregnancy and in the non-BIO group, csDMARDs were only used before pregnancy. One case was administered PSL after pregnancy in the BIO group; other cases were administered PSL before pregnancy. After confirming the pregnancy, BIO agents and csDMARDs were withdrawn in all cases.

	Cases	Age at pregnancy (years)	Diagnosis	Disease duration (years)	Steinbrocker staging			Medication		Dose of PSL during pregnancy (mg/day)	Using NSAIDs during pregnancy
					Stage	Class	Before pregnancy	During pregnancy	After delivery		
BIO group	1a	33	RA	2	2	2	ADA, PSL	PSL	ADA, MTX, PSL	18	+
	1b	36	RA	5	2	2	ETN, PSL	PSL	ETN, PSL, NSAIDs	14	+
	2a	33	RA	3	2	3	ETN, PSL	PSL	MTX, PSL	5	+
	2b	35	RA	5	2	3	ETN, PSL	PSL	ETN, PSL	10	+
	3	32	JIA	20	4	2	TCZ	PSL	TCZ, PSL	5	+
	4	29	RA	9	4	2	ETN, PSL	PSL	PSL	5	+
	5	31	JIA	18	4	3	ETN	-	NSAIDs	-	-
	6a	27	RA	5	3	2	ETN, PSL	PSL	ETN, MTX, PSL	7.5	-
	6b	31	RA	9	3	2	TCZ, SASP, PSL	PSL	TCZ , MTX	10	+
	7a	32	RA	6	2	1	ETN	-	ETN, MTX	-	-
	7b	34	RA	8	2	1	ETN	-	ETN, MTX	-	-
	8	31	RA	7	3	2	ETN, BUC, PSL	PSL	ETN, SASP, PSL	5	+
	9	25	JIA	9	4	1	ETN, SASP	-	ETN, MTX	-	-
Sub-total	9 women, 13 pregnancies	31.5 (25-36)	6 RA, 3 JIA	8.2 (2-20)	2-6, 3-3, 4-4	1-3, 2-7, 3-3		69% (9/13)		8.8 (5-18)	62% (8/13)
Non-BIO group	10	29	RA	2	3	2	SASP	-	MTX, SASP, PSL	-	-
	11	30	RA	3	3	3	SASP, PSL	PSL	MTX, SASP, NSAIDs	6	-
	12	35	RA	0.8	4	2	SASP	-	CZP, PSL	-	-
	13	39	RA	0.6	1	1	PSL	PSL	-	5	-
	14	33	RA	9	3	1	PSL	PSL	ETN, SASP, PSL	10	-
	15a	28	JIA	12	4	2	BUC	-	-	-	-
	15b	30	JIA	14	4	2	-	-	-	-	-
Sub-total	6 women, 7 pregnancies	32 (28-39)	5 RA, 1 JIA	5.9 (0.6-14)	1-1, 3-3, 4-3	1-2, 2-4, 3-1		43% (3/7)		7 (5-10)	0% (0/7)
Total	15 women, 20 pregnancies	31.7 (25-39)	11 RA, 4 JIA	7.2 (0.6-20)	1-1, 2-6, 3-6, 4-7	1-5, 2-11, 3-4		57% (12/21)		8.4 (5-18)	38% (8/21)

**Table 2:** Characteristics of pregnancy and delivery in BIO and non-BIO groups in patients with rheumatoid arthritis BIO: biologics, csDMARD: conventional synthetic disease-modifying antirheumatic drug In the BIO group, biologics and/or csDMRADs were used before pregnancy and in the non-BIO group, csDMARDs were only used before pregnancy.

	Cases	Gestational age (weeks, days)	Preterm birth	Birth-weight (g)	Low-birth-weight infant	Caesarean section
BIO group	1a	39w		2340	+	
	1b	36w	+	2350	+	
	2a	33w6d	+	1990	+	+
	2b	38w1d		2938		+
	3	37w5d		2678		
	4	37w5d		2180	+	
	5	37w2d		2678		
	6a	36w	+	2406	+	
	6b	35w	+	2100	+	
	7a	39w		2906		
	7b	38w		2578		
	8	40w		3014		
	9	40w		3056		
Sub-total	9 women, 13 pregnancies	37.6w	31% (4/13)	2555 (1990-3056)	46% (6/13)	15% (2/13)
Non-BIO group	10	37w		2978		
	11	36w	+	2400	+	+
	12	38w3d		3400		
	13	34w5d		Fetal death		
	14	39w		2562		
	15a	40w		2976		
	15b	40w		3060		
Sub-total	6 women, 7 pregnancies	37.9w	14% (1/6)	2896 (2400-3400)	33% (1/6)	17% (1/6)
Total	15 women, 20 pregnancies	37.7w	5 preterm births, 35.4 weeks, 26% (5/19)	2663 g (1990-3400), 1 fetal death	2252 g (1990-2406) 37% (7/19)	16% (3/19)

**Table 3:** CDAI before and after pregnancy CDAI: Clinical Disease Activity Index, BIO: biologics *p<0.05, Mann-Whitney U test

	Prepregnancy CDAI	Postdelivery CDAI	p
BIO group	9.3±1.8	19.9±1.8	0.002*
Non-BIO group	16.0±3.8	17.9±2.4	0.7
Total	12.0±1.8	19.2±2.7	0.008*

**Figure 1:**
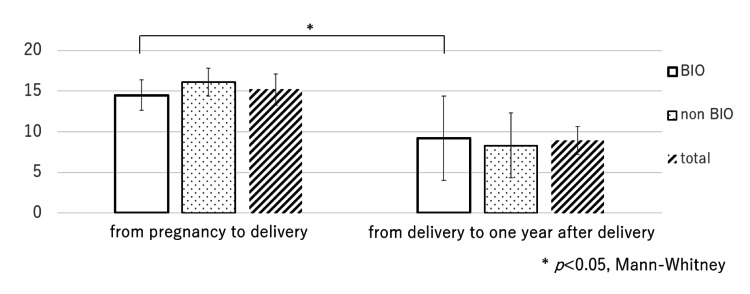
ΔmTSS from pregnancy to delivery and from delivery to one year later in BIO and non-BIO groups mTSS: modified total Sharp score, BIO: biologics ΔmTSS values from prepregnancy to postdelivery (mean duration, 10 months (7-11 months)) and from delivery to one year after delivery (mean duration, 12 months (7-18 months)) were 14.5±4.8 and 9.2±1.7 in the BIO group (white square, *p*<0.05), respectively, 16.1±14.8 and 8.3±10.5 in the non-BIO group (dotted square), respectively, 15.2±14.0 and 8.9±9.0 in total (hatched-pattern square), respectively. Bone and joint destruction in both groups progressed during the perinatal period including even one year after delivery (not statistically significant between BIO and non-BIO groups), although conventional synthetic disease-modifying antirheumatic drugs (csDMARDs) and/or BIO were readministered after 15 deliveries in 11 cases.

ΔmTSS values from prepregnancy to postdelivery and from delivery to one year after delivery were 14.5±4.8 and 9.2±1.7 in the BIO group (*p*<0.05), 16.1±5.2 and 8.3±4.0 in the non-BIO group, 15.2±1.9 and 8.9±1.7 in total (Figure 1). Bone and joint destruction in both groups progressed during the perinatal period including even one year after delivery, although csDMARDs and/or BIO were readministered after 15 deliveries in 11 cases.

Next, we investigated the CDAI and ΔmTSSs in pregnant patients with seven low-birth-weight infants compared to pregnant patients with 13 normal-birth-weight infants. Seven low-birth-weight infants were two full-term and five preterm births. On the other hand, 13 normal-birth-weight infants were all full-term births. CDAI values in pregnant patients with low-birth-weight infants were higher compared to pregnant patients with normal-birth-weight infants at pregnancy (13±3.0 vs. 11.6±0.6, *p*<0.05) and tended to be high at delivery (21±3.6 vs. 18.2±1.8, *p*=0.06). ΔmTSSs from prepregnancy to postdelivery and from delivery to one year after delivery in pregnant patients with low-birth-weight infants tended to be higher compared to pregnant patients with normal-birth-weight infants at pregnancy (19.4±5.9 vs. 13.5±3.1, *p*=0.52, and 13.4±2.1 vs. 7.0±2.5, *p*=0.06, respectively; Table 4).

**Table 4:** Comparison between pregnant patients with low-birth-weight infants and with normal-birth-weight infants CDAI: Clinical Disease Activity Index, mTSS: modified total Sharp score *p<0.05, Mann-Whitney U test

		With low-birth-weight infants	With normal-birth-weight infants	p
CDAI	At pregnancy	13±3.0	11.6±0.6	<0.05*
	At delivery	21±3.6	18.2±1.8	0.06
ΔmTSS	From prepregnancy to postdelivery	19.4±5.9	13.5±3.1	0.52
	From delivery to one year after delivery	13.4±2.1	7.0±2.5	0.06

## Discussion

In this study, the disease activity worsened, and bone and joint destruction progressed during the perinatal period in both the BIO and non-BIO groups in patients with RA and JIA. One year after delivery, bone and joint destruction was observed to continue even after resuming drug therapy for RA and JIA. In addition, of 19 childbirths, there were 5 preterm births (26%) and 7 low-birth-weight infants (37%).

Withdrawal of antirheumatic drugs in patients with RA and JIA can lead to recurrence of disease activities [3,4,7,9,11]. In addition, if the disease is active during pregnancy, it may not only progress to joint destruction but also adversely affect pregnancy and pregnancy continuation [7,10]. High disease activity during gestation has been reported as a risk factor for preterm birth. It is important to maintain low disease activity in pregnant patients to prevent arthritis and promote a safe pregnancy [3,4,7,11]. Before the approval to use some csDMARDs and biologics for perinatal patients with RA, patients with active RA who hope to become pregnant and have a child are forced to make a choice between maintaining strict RA treatment or pregnancy with DMARD discontinuation. If a woman chooses pregnancy, she must stop taking most antirheumatic drugs. Therefore, the disease activity of RA is worse because treatment is stopped or the patient is switched to less aggressive therapy, and joint destruction and dysfunction progress. In addition, the worse disease activity of RA causes infertility, miscarriage, fetal death and low-birth-weight infants [2-4,9]. In this study, the preterm birth rate was higher in the BIO group compared with the non-BIO group (31% vs. 14%, not significant), and the low-birth-weight infant rate was also increased (46% vs. 33%, not significant). In the general population, the preterm birth rate was 5.7%, and the low-birth-weight infant rate was 9.6% in Japan [9,15]. In our case series, the rates of the two parameters were greater than those of the general population, especially in the BIO group. This result may indicate that the patients in the BIO group had more severe RA disease activity. A fetal death occurred in non-BIO group. Smeele and Dolhain reported that women with RA are at an increased risk of small-for-gestational-age (SGA) infants (the risk being 10% in RA patients vs. 3% in the general population), intrauterine growth restriction (twofold higher compared with the general population), preterm birth, and cesarean section than the general population [16]. It has also been reported that patients with RA with a high activity phase of disease in early pregnancy tend to have a higher frequency of preterm birth and low-birth-weight infants [9,15]. In fact, the pregnant patients with low-birth-weight infants were related to the higher disease activity and tended to have greater joint destruction compared to the pregnant patients with normal-birth-weight infants in this study.

According to the current perinatal treatment guidelines for patients with RA, it is important to continue safe drugs during the perinatal period to keep the disease in remission and suppress the progression of disease activity for establishing and maintaining safe pregnancy. In the case of patients with RA who desire to bear children, physicians should consider withdrawing contraindicated drugs during pregnancy and use drugs safe for the fetus or infant during pregnancy and breastfeeding. If the contraindicated drugs must be continued due to the patient’s high disease activity, informed consent should be obtained from the patient regarding the risks and benefits of continuing their drugs. Effective and safe drugs that are permitted for use during the perinatal period should be continued [3,4,7,9].

In this study, BIO and csDMARDs were withdrawn after confirming pregnancy. PSL was administered in 64% cases in the BIO group and 43% of cases in the non-BIO group. In both groups, withdrawal of BIO and csDMARDs during pregnancy led to recurrence of the disease activity and progression of joint destruction. The progression of joint destruction was slower after delivery but was steady even after drugs were resumed. Maternal anti-TNF treatment is associated with an increased risk of pediatric infections for the first three years [17]. Although anti-TNF agents were associated with increased risks of preterm birth, cesarean section, and SGA infants; these risks may be associated with disease activity of RA and no agent-specific effects [17]. In this study, no pediatric infection was noted in our cases one year after delivery.

van Dunné et al. reported that a high rate of joint damage in patients with RA two years after starting drug therapy was associated with a history of miscarriage but not with fecundity. They discussed the reason why a predominant Th2 response was necessary for normal pregnancy, but an inborn predominance of the Th1 response in the miscarriage subgroup affected both the disease process in the joint and the physiological immunological changes during pregnancy [18]. Based on the above, we support the adverse effects on the mother and fetus due to the disease activity recurrence of RA and recommend continuing or resuming BIO or csDMARDs with the consent of the pregnant patient if the benefits of using the drugs outweighed the risk [2-4,7,9,11].

To the best of our knowledge, no previous studies have evaluated bone and joint destruction according to the use of mTSS values in adult patients with RA and JIA in the perinatal period. However, there are some limitations in this study. First, the sample size of the pregnant patients with RA and JIA was relatively small. Second, X-ray examination was not able to be performed at the same point in all cases because this retrospective study did not have a protocol for the schedule of the examinations. Especially, the examination point at prepregnancy might vary wildly from the accurate time of their pregnancy; however, it seems difficult to estimate and set it correctly in real world. Third, ultrasound imaging or magnetic resonance imaging was not used in this study. Imaging modalities, which are permitted to be used during pregnancy, should be used in the future study. Fourth, no cases continued to use BIO and/or csDMARDs, including SASP and TAC, which were allowed to take even during the organogenesis period in the perinatal period. However, most cases continued or began PSL. It is necessary to prospectively evaluate the bone and joint destruction in the BIO continuation group and the non-BIO group throughout the perinatal period with large populations in multiple centers in a future study.

## Conclusions

During the perinatal period in the adult patients with RA and JIA, their disease activity worsened with the progression of bone and joint destruction in both the BIO and non-BIO groups in this study. This result suggests that it is important to control the disease activity of RA and JIA, and prevent bone and joint destruction by continuing drug therapy with DMARDs, even BIO, that can be administered during the perinatal period.
